# CRL4 ubiquitin ligase stimulates Fanconi anemia pathway-induced single-stranded DNA-RPA signaling

**DOI:** 10.1186/s12885-019-6305-x

**Published:** 2019-11-05

**Authors:** Tamara Codilupi, Doreen Taube, Hanspeter Naegeli

**Affiliations:** Institute of Pharmacology and Toxicology, University of Zurich-Vetsuisse, Winterthurerstrasse 260, 8057 Zurich, Switzerland

**Keywords:** Chemotherapy, Cisplatin, Crosslink - CUL4 - Fanconi anemia, ssDNA

## Abstract

**Background:**

DNA-crosslinking agents like cisplatin and mitomycin C (MMC) are indispensible for the treatment of many solid malignancies. These anticancer drugs generate DNA interstrand crosslinks (ICLs) that cause cell death by blocking replication forks. Many factors counteracting ICL-induced DNA replication stress, including the Fanconi anemia (FA) pathway, are regulated by ubiquitination and, therefore, ubiquitin ligases are potential targets for the sensitization of cancer cells to crosslinking agents. In this study, we investigated the function of the CRL4 ubiquitin ligase in modulating the response of cancer cells to ICL induction.

**Methods:**

The two cullin paralogs CUL4A and CUL4B, which form the CRL4 ligase scaffold, were depleted in cancer cells by small interfering RNA followed by analysis of the cellular and biochemical responses to ICLs elicited upon cisplatin or MMC treatment.

**Results:**

We report that the combined depletion of CUL4A and CUL4B weakens an FA pathway-dependent S phase checkpoint response. CRL4 positively stimulates the monoubiquitination of FANCD2 required for the recruitment of XPF-ERCC1, a structure-specific endonuclease that, in turn, contributes to the display of single-stranded DNA (ssDNA) at ICLs. After CRL4 down regulation, the missing ssDNA results in reduced recruitment of RPA, thereby dampening activation of ATR and CHK1 checkpoint kinases and allowing for S phase progression despite ICL induction.

**Conclusion:**

Our findings indicate that CRL4 promotes cell survival by potentiating an FA pathway-dependent ssDNA-RPA signaling platform installed at ICLs. The anticancer efficacy of crosslinking agents may, therefore, be enhanced by down regulating CRL4 activity.

## Background

Platinum- and mitomycin-based drugs are used against solid malignancies including lung, bladder, esophageal, testicular, ovarian and cervical cancer [1]. The mechanism of action of cis-diamminedichloroplatinum (II) (cisplatin) and mitomycin C (MMC) involves the formation of DNA interstrand crosslinks (ICLs), which lead to cell death primarily by interfering with DNA replication [2]. A common cause of treatment failure is the emergence of resistance developing in most patients even after an initially favorable response. Cancer cells avoid ICL-induced cytotoxicity by eliciting the DNA damage response (DDR), which coordinates cell cycle progression with DNA repair [3, 4]. A universal DDR trigger is DNA replication stress involving persistent stretches of single-stranded DNA (ssDNA) at stalled replication forks. The locally arising ssDNA is rapidly coated by replication protein A (RPA), thus forming ssDNA-RPA complexes that provide a platform for engagement of the ataxia telangiectasia-mutated and Rad3-related (ATR) kinase. This serine/threonine kinase phosphorylates RPA, as well as signaling intermediates like checkpoint kinase 1 (CHK1) and histone H2AX, to trigger cell cycle checkpoints [5, 6]. The efficiency of checkpoint activation determines how cancer cells respond to chemotherapy [7, 8] and, accordingly, RPA hyperphosphorylation has been linked to increased cisplatin resistance [9].

The DDR cascade is driven by posttranslational modifications involving, besides phosphorylation, polypeptide modifiers like ubiquitin [10, 11]. Cullin-RING ubiquitin ligases (CRLs) contain a cullin scaffold (CUL1 to 5, CUL7 or CUL9) that recruits substrate receptors to target proteins for ubiquitination [12–15]. CRL activation may require modification of cullin subunits by the ubiquitin-like modifier NEDD8 [16]. MLN4924 (pevonedistat) is a small-molecule antagonist of this neddylation reaction, thereby inhibiting CRLs and preventing the ubiquitination and subsequent degradation of proteins [17]. A prominent target of CRL-mediated degradation under replication stress is the replication-licensing factor CDT1, whose function is to initiate replication forks. Normally, only one round of DNA synthesis is allowed during each cell cycle [14, 18]. However, by preventing the ubiquitination and proteasomal degradation of CDT1, MLN4924 induces the superfluous initiation of extra replication forks, causing aberrant DNA re-replication [15, 19, 20].

Previous reports demonstrated that MLN4924 also sensitizes cancer cells to the cytotoxic action of cisplatin and MMC [21–23], implying that CRL inhibitors may mitigate resistance against crosslinking agents. However, the mechanism of this synergy between MLN4924 and crosslinking drugs remained unclear. It was not known which of the many possible CRLs susceptible to inhibition by MLN4924 are implicated in the response to DNA-crosslinking agents and, in particular, it was not known how CRLs affect the detection or signaling of DNA damage inflicted by these drugs. Here, we identified CRL4 as an additional player modulating the cellular sensitivity to cisplatin and MMC, and found that the cullin paralogs CUL4A and CUL4B display redundant functions in regulating cell survival after treatment with crosslinking agents. The concomitant down regulation of these exchangeable CUL4 scaffolds diminishes the Fanconi anemia (FA) pathway-dependent recruitment of XPF-ERCC1, which as part of a nuclease complex contributing to the formation of ssDNA at ICL sites. Accordingly, this CRL4 depletion interferes with the assembly of ssDNA-RPA intermediates upon cisplatin or MMC treatment, such that activation of ATR and the phosphorylation of RPA, CHK1 and H2AX are reduced. Our results indicate that CRL4 activity protects from cancer cell death after treatment with crosslinking agents by stimulating an FA pathway-induced S phase checkpoint.

## Methods

### Cell lines and treatment

HeLa (catalog designation CCL-2) and SKOV3 cells (catalog designation HTB-77) were purchased from ATCC and cultured in low-glucose Dulbecco’s modified Eagle medium (DMEM) and Roswell Park Memorial Institute (RPMI) 1640 medium, respectivel. Cell culture media (obtained from Gibco) were supplemented with 10% (v/v) fetal calf serum and 100 U/ml penicillin-streptomycin. All cells were recently tested negative for mycoplasma contamination and authenticated by short tandem repeat profiling (Microsynth). Cells were incubated at 37 °C in a humidified atmosphere under 5% CO_2_. The cisplatin (Sigma) solutions were prepared freshly each time in DMEM. MMC (Sigma) was dissolved as a 1.5-mM stock solution in phosphate-buffered saline (PBS) and MLN4924 (ApexBio) as a 50-mM stock solution in dimethyl sulfoxide (DMSO) and further diluted in cell culture medium. Cells were treated with crosslinking agents 3 days after siRNA transfections, except for the viability assays where the drugs were applied 2 days after transfections.

### siRNA transfections

Transfections were performed with Lipofectamine RNAiMAX (Invitrogen) according to the manufacturer’s protocol. All siRNA sequences are shown in the Additional file 1: Table S1. The siRNA concentrations were 24 nM except for siDDB1, which was used at a concentration of 8 nM.

### Cell viability

Resazurin was purchased from Alfa Aesar and viability measured according to the manufacturer’s instruction. Briefly, 2000 cells per well were seeded into a 96-well plate and 24 h later treated with the indicated drug concentrations. Following 2 days, resazurin was added to the cells and fluorescence measured after 3 h (LS55 luminescence Spectrometer; Perking Elmer). Cell viability was expressed as the percentage of controls obtained in the absence of cisplatin and IC_50_ values were calculated using GraphPad Prism.

### Cytotoxicity

Cell death was measured using the LDH Cytotoxicity Assay Kit (Pierce). Briefly, 5000 cells per well were seeded into a 96-well plate. After 24 h, cells were treated with increasing concentrations of cisplatin for 2 days and the released LDH was measured in the supernatant according to the manufacturer’s instruction. Results are calculated as the ratio of released LDH in relation to maximal LDH activity in each condition, and expressed as the percentage of the ratios detected with untreated controls.

### Colony formation

Cell survival was performed as described [24]. Briefly, cells were treated with increasing concentrations of cisplatin for 2 h, extensively washed with PBS and further incubated in fresh media without drug for 10 days. Colonies were fixed and stained with 0.25% (w/v) crystal violet solved in 80% (v/v) ethanol. Colonies composed of at least 50 cells were counted and surviving fractions were normalized to untreated controls.

### Immunoblotting

Cells were treated as indicated, washed once with PBS and lysed in RIPA buffer [50 mM Tris-HCl, pH 7.5, 1% (v/v) NP-40, 0.5% (w/v) sodium deoxycholate, 0.1% (w/v) SDS, 150 mM NaCl, 2 mM EDTA] complemented with 1 mM N-ethylmaleimide (NEM, Sigma), 1 mM phenylmethylsulfonyl fluoride, PhosStop (Roche) and Complete Protease Inhibitor cocktail (Roche) for 10 min on ice. After sonication for 5 cycles (30 s on, 30 s off) at 4 °C (Biorupture Plus; Diagenode), protein concentration was determined by the BCA protein assay (Pierce) according to the manufacturer’s instruction. Laemmli buffer was added and boiled for 5 min at 98 °C; 10 μg of protein were separated on 4–20% Criterion TGX stain-free precast gels and transferred to nitrocellulose membranes using a Turbo transfer device (Bio-Rad). Membranes were incubated with primary antibodies (Additional file 1: Table S2) over night at 4 °C followed by incubation with fluorescence-labelled secondary antibodies for 30 min. Membranes were developed using the Odyssey CLx Imaging System and quantification of protein expression was performed using the Image Studio Lite Software (Li-Core Biosciences).

### Cell cycle analysis

Replicative cells were labelled for 3 h with 5-ethynyl-2′-deoxyuridine (EdU, Sigma) and fixed in 1% (w/v) paraformaldehyde for 10 min. Coupling of Alexa Fluor 488 was performed using the Click-iT EdU Flow Cytometry Assay Kit (Invitrogen) according to the manufacturer’s instruction. DNA contents were quantified by 4′,6-diamidino-2-phenylindole (DAPI) staining. Mitotic cells were visualized by incubation with the phospho-histone H3 (pH 3) antibody (Additional file 1: Table S2) for 2 h, followed by a 1-h secondary antibody incubation using anti-mouse Alexa 647. Approximately 10,000 and 50,000 cells per sample were analyzed for EdU and pH 3, respectively, using a Fortessa LSR ll flow cytometer followed by data analysis using the FlowJo software.

### Immunofluorescence microscopy

Cells were grown on glass coverslips and treated as indicated 3 days after siRNA transfections. Following the indicated incubation periods, cells were washed with PBS and pre-extraction buffer [25 mM HEPES, pH 7.5, 50 mM NaCl, 1 mM EDTA, 3 mM MgCl_2_, 300 mM sucrose, 0.5% (v/v) Triton X-100] was added for 2 min [25]. Cells were fixed with 4% (w/v) paraformaldehyde in PBS for 10 min, followed by incubation with PBS containing 0.2% (v/v) Triton X-100 and 3% (w/v) bovine serum albumin (BSA) for 10 min. Coverslips were then washed with 1% BSA in PBS and incubated with primary antibodies (Addional file 1, Table S2) diluted with 1% BSA in PBS. Secondary antibodies, diluted with 1% BSA in PBS and containing DAPI were added for 30 min at 37 °C after washing three times for 10 min with 1% BSA in PBS. Images of immunostained cells were taken with an SP8 confocal microscope (Leica) and analysed with the ImageJ software.

### Monitoring of ssDNA

To detect ssDNA, cells were labelled with 25 μM 5-iodo-2′-deoxyuridine (IdU, Sigma) 30 h prior to anticancer drug treatment as described [5]. The ssDNA was detected using an anti-IdU antibody (BD Biosciences) under native conditions. To quantify the totally incorporated IdU, DNA was denaturated with 2 M HCl in 0.5% (v/v) Tween 20 for 40 min and washed twice with 0.1 M Na-borate buffer, pH 9.0, prior to antibody staining. Images taken with an SP8 confocal microscope (Leica) were analysed using the ImageJ software.

### Quantitative reverse transcriptase-PCR (qRT-PCR)

To determine the knock down efficiency, mRNA was extracted 3 days after siRNA transfection using the RNeasy Mini Kit (Qiagen) according to the manufacturer’s instructions. Thereafter, cDNA was synthesized from 500 ng mRNA with the iScript cDNA synthesis kit from Bio-Rad. Gene specific primers were designed using the NCBI Primer-BLAST [26] and GAPDH served as the internal control (Additional file 1: Table S1). Quantitative PCR was performed using the KAPA SYBR Fast qPCR Master Mix (2x) kit (KAPA Biosystems) according to the manufacturer’s instructions. The amplification conditions in the Bio-Rad CFX instrument consisted of an initial step of 3 min at 95 °C followed by 40 cycles of 3 s at 95 °C and 40 s at 60 °C. The delta-delta ct method was used to determine relative mRNA expression levels between siRNA-transfected samples and control samples transfected with non-coding siRNA [27].

### In vitro protein dephosphorylation

HeLa cells were harvested 3 days after siRNA transfections and lysed for 30 min on ice under mild lysis conditions [1% (wt/vol) NP-40, 0.5% (wt/vol) SDS, complete protease inhibitor cocktail, EDTA-free (Roche)] followed by sonication for 10 cycles (30 s on, 30 s off) at 4 °C (Biorupture Plus, Diagenode). Cell lysates were then diluted in CIP buffer (100 mM NaCl, 50 mM Tris-HCl, pH 8.0, 10 mM MgCl_2_, 1 mM DTT, complete protease inhibitor cocktail - EDTA-free) and complemented with calf intestinal alkaline phosphatase (2 U/μg of protein, Sigma), and / or PhosStop (Roche) and / or 1 mM N-ethylmaleimide (NEM, an inhibitor of deubiquitinases) [28]. Reactions were incubated for 2 h at 37 °C, boiled in Laemmli buffer for 5 min and subjected to Western blot analysis.

### Statistical analyses

GraphPad Prism 5 was used to perform statistical analyses. The data presented were acquired from a minimum of two independent experiments. The Student’s *t*-test (unpaired, two-tailed) was used to analyze immunoblot and flow cytometry assays and all data are shown as the mean ± SEM. Immunofluorescence microscopy experiments were analyzed using 1-way ANOVA according to Kruskal-Wallis. Median values were presented as horizontal lines, boxes show the upper and lower quartiles and whiskers the 10-90th percentiles. *P* values of *P < 0.05, **P < 0.01 and ***P < 0.001 were considered to indicate statistical significance.

## Results

### CUL4A/B depletion potentiates the cytotoxicity of crosslinking agents

**Fig. 1:**
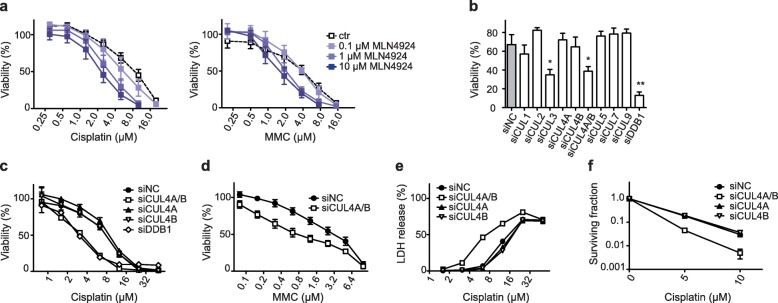
CUL4A/B depletion potentiates ICL cytotoxicity. **a** HeLa cells were incubated for 48 h with cisplatin (panel on the left) or MMC (panel on the right) together with MLN4924 as indicated (*N* = 5–10 experiments, error bars show s.e.m.). Cell viability is given as the percentage of controls not exposed to cisplatin. **b** HeLa cells were transfected with indicated siRNA, incubated with 5 μM cisplatin and tested after 48 h. Viability is expressed as the percentage of control values obtained in the absence of cisplatin (*N* = 3–5); siNC, non-coding RNA control. Asterisks indicate significantly lower viability in depleted cells relative to non-coding controls (*P < 0.05 and **P < 0.01, unpaired two-tailed t-test). **c** Cells were transfected with the indicated siRNAs, incubated with cisplatin and tested for viability after 48 h (N = 5). **d** Cell viability after exposure to MMC (*N* = 5). **e** Cytotoxicity assays measuring the release of LDH from siRNA-transfected cells during 48-h treatments with cisplatin (N = 5–10). **f** Colony-forming assays after exposure of siRNA-transfected cells to the indicated cisplatin concentrations. The resulting colony numbers are normalized to non-exposed controls (*N* = 5)

We started out with short-term viability assays, based on the cell-mediated resazurin reduction, to establish that the neddylation inhibitor MLN4924 potentiates the cytotoxic effect of the crosslinking agents cisplatin and MMC in HeLa cells, as demonstrated before with several other cancer cell lines [21, 22]. MLN4924 at a concentration of 10 μM reduces the IC_50_ of cisplatin from ~ 10 to ~ 2.5 μM and the IC_50_ of MMC from ~ 4 to ~ 1.5 μM (Fig. 1a). MLN4924 also increases the cytotoxicity of cisplatin and MMC in SKOV3 ovarian carcinoma cells (Additional file 1: Figure S1a and S1b).

Next, we depleted different cullins by siRNA transfections to understand which of the possible cullin targets of neddylation modulates this vulnerability to DNA-crosslinking agents. Cell viability assays, carried out in the presence of 5 μM cisplatin, confirmed a potentiation of cisplatin toxicity upon down regulation of CUL3 as reported before for SKOV3 and ES2 ovarian carcinoma cells [29]. The new finding of this screen is that a sensitization to cisplatin cytotoxicity is also detected upon simultaneous down regulation of the two scaffold paralogs of CRL4, i.e., CUL4A and CUL4B (Fig. 1b). Dose dependence experiments showed that this co-depletion of CUL4A and CUL4B mimics to a considerable extent the sensitizing effect of MLN4924 when cells are treated with cisplatin or MMC for 48 h (Fig. 1c and d). Nearly the same increase of sensitivity to cisplatin was achieved upon depletion of the CRL4 adaptor protein Damaged DNA-binding 1 (DDB1) instead of the CUL4A/B scaffold. Instead, no sensitization was elicited upon individual depletion of only one of the cullins, CUL4A or CUL4B, indicating that the two interchangeable scaffolds have a redundant function. These results were confirmed using distinct combinations of siRNA sequences targeting CUL4A and CUL4B to exclude off-target effects (Additional file 1: Figure S1c and S1d). The efficiency of protein down regulation upon siRNA transfections is documented in Additional file 1: Figure S2.

Further assays measuring the release of lactate dehydrogenase as a marker of membrane disruption (Fig. 1e) confirmed that the CUL4A/B depletion enhances cisplatin-induced cell death. Finally, the increased cytotoxicity of cisplatin upon a combined CUL4A/B depletion, but not after down regulation of only one of the cullins individually, was confirmed in a long-term colony-forming assay (Fig. 1f).

### CUL4A/B depletion reduces H2AX/RPA phosphorylation upon ICL induction

**Fig. 2:**
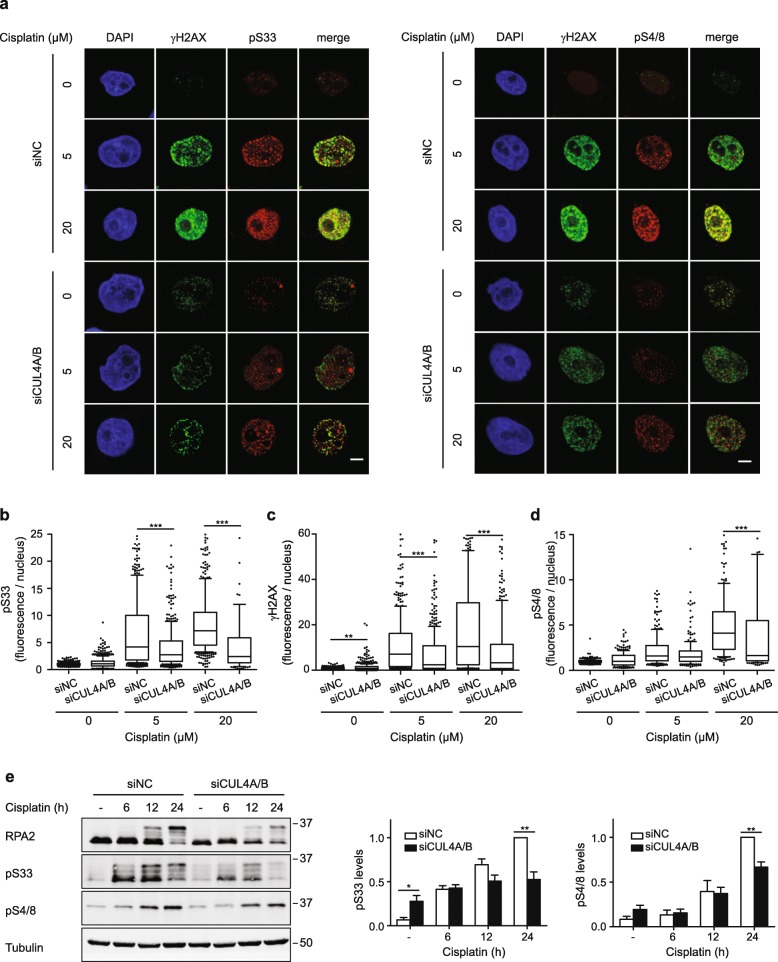
CRL4 depletion reduces H2AX and RPA phosphorylation upon cisplatin exposure. **a** HeLa cells were transfected with siRNA and subjected to 24-h cisplatin treatments as indicated. For the detection of γH2AX, pS33 and pS4/S8 (RPA2 phosphorylated at serine 33 and 4/8, respectively), fixed cells were stained with phospho-specific antibodies. DAPI visualizes the nuclei. **b** Quantification of nuclear fluorescence representing pS33 induced by the indicated treatments (*N* = 210–580 nuclei from 2 to 4 experiments). **c** Quantification of γH2AX (*N* = 510–590 nuclei from 3 to 4 experiments). **d** Quantification of pS4/8 foci (*N* = 180–250 nuclei from 2 experiments). In panels b, c and d, horizontal lines represent median values, boxes 25-75th percentiles and whiskers 10-90th percentiles. ***P < 0.001 (1-way ANOVA according to Kruskal-Wallis). Scale bar: 10 μm. **e** HeLa cells were transfected with siCUL4A/B, or with siNC, incubated with 5 μM cisplatin and analyzed following the indicated periods. Whole cell lysates were probed with antibodies against RPA2, pS33 and pS4/8. Tubulin served as the loading control. The graphs represent quantifications of pS33 and pS4/8, normalized to tubulin, from 3 to 5 experiments. All values are shown relative to the respective phospho-protein observed in siNC-transfected cells after a 24-cisplatin treatment. Asterisks indicate significant difference between CUL4A/B-depleted cells and non-coding controls (*P < 0.05, **P < 0.01; unpaired, two-tailed t-test)

Considering that CUL4A and CUL4B have an impact on the cytotoxicity of crosslinking agents, we tested the role of CRL4 in modulating DNA damage signaling following ICL induction. Resolution of ICLs by the FA pathway generates transient DNA breaks and ssDNA intermediates, which activate the checkpoint kinases ATR and ATM [5, 8, 30–32]. Phosphorylation of downstream factors like histone H2AX and the ssDNA-binding protein RPA generates docking motifs for effectors that mediate S phase checkpoints essential for DNA repair [3, 33–35]. Phosphorylation of H2AX (generating γH2AX) as well as phosphorylation of RPA2, the middle subunit of RPA, on serines 4/8 (generating pS4/8) and serine 33 (generating pS33), was assessed by immunofluorescence using phospho-specific antibodies. CRL4-proficient cells respond to cisplatin treatment with a dose-dependent increase of γH2AX, pS4/8 and pS33, but this phosphorylation was markedly reduced for the direct ATR targets pS33 and γH2AX (Fig. 2a-c) in CUL4A/B-depleted cells. A significant reduction was also observed in CUL4A/B-depleted cells for the formation of pS4/8, but only at the highly cytotoxic cisplatin concentration of 20 μM (Fig. 2d).

**Fig. 3:**
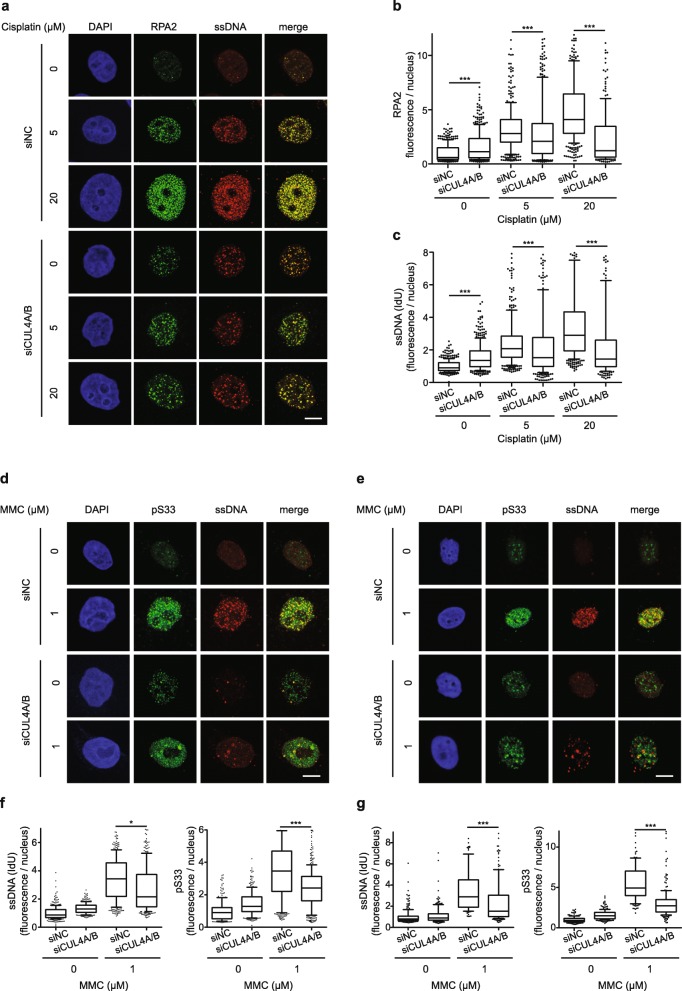
CRL4 deficiency impairs the interstrand crosslinks dependent assembling of ssDNA-RPA complexes. **a** HeLa cells were transfected with the indicated siRNA and labeled with IdU prior to cisplatin exposure for 24 h. After fixation, cells were stained for RPA2 and ssDNA. DAPI was used to visualize the nuclei. **b** Quantification of nuclear fluorescence representing RPA2 foci (*N* = 360–680 nuclei from 3 to 6 experiments). **c** Quantification of nuclear fluorescence representing ssDNA induced by the indicated treatments (*N* = 230–530 nuclei from 3 to 4 experiments). **d** HeLa cells were transfected with siRNA and labeled with IdU prior to MMC exposure for 24 h. For the detection of pS33 (RPA2 phosphorylated at Ser33), fixed cells were stained with phospho-specific antibodies. DAPI was used to visualize the nuclei. **e** SKOV3 cells were transfected with siRNA as indicated, treated for 24 h with MMC, and stained for pS33 and ssDNA. **f** Quantification of nuclear fluorescence representing ssDNA and pS33 foci in HeLa cells (*N* = 262–349 nuclei from 2 experiments). **g** Quantification of ssDNA and pS33 foci in SKOV3 cells (*N* = 200–240 nuclei from 2 experiments). In panels **b**, **c**, **f**, **g**, horizontal lines represent median values, boxes 25-75th percentiles and whiskers 10-90th percentiles. *P < 0.05, ***P < 0.001 (1-way ANOVA according to Kruskal-Wallis). Scale bar: 10 μm

The phosphorylation of RPA2 at serines 4, 8 and 33 was further investigated by immunoblotting (Fig. 2e). Some increase of RPA2 phosphorylation was observed in CUL4A/B-deficient cells without any genotoxic treatment. This response is expected from the loss of CRL4-dependent licensing regulation (see Discussion). In blots with the generic RPA2 antibody, there was an apparent reduction of the overall RPA2 signal in CRL4-deficient cells compared to the CRL4-proficient counterparts. However, we did not find in the literature any indication that CRL4 would positively regulate RPA stability, such that a CRL4 down regulation could result in diminished RPA2 levels. We favor the view that the higher background phosphorylation of RPA2 in CRL4-deficient cells results in a reduced signal intensity in the electrophoretic position corresponding to the unmodified protein. This interpretation is supported by the immunofluorescence analyses (with quantifications) of Fig. 3, where in the absence of any crosslinking agent there is no reduction of RPA2 in CRL4-deficient cells compared to controls. When using phospho-specific antibodies, the immunoblots of Fig. 2e confirmed the observed reduction of pS4/8 and pS33 in CUL4A/B-depleted cells compared to CRL4-proficient counterparts after 24-h cisplatin exposures This finding was validated using a second siRNA sequence for the down regulation of CUL4A and CUL4B (Additional file 1: Figure S3a and S3b). To ensure that the observed shift in RPA2 electrophoretic mobility results from phosphorylation and not from a hypothetical ubiquitination by CRL4, cell lysates were subjected to phosphatase incubation prior to immunoblotting. Such a dephosphorylation clearly diminished the pS33 signal, thus confirming that we detected truly phosphorylated RPA2 (Additional file 1: Figure S3c). These results indicate that CUL4A/B-deficient cells are impaired in DNA damage signaling following cisplatin treatment.

### CRL4 deficiency impairs interstrand crosslink-dependent assembly of ssDNA-RPA complexes

In view of the observation that the cisplatin-dependent RPA phosphorylation is reduced in CRL4 deficient cells, we next tested whether CRL4 is required for the recruitment of RPA to sites of cisplatin damage. By immunofluorescence analysis, some increase of RPA2 foci in chromatin was observed in CUL4A/B-deficient cells even without any genotoxic treatment (Fig. 3a and b). This response is expected from the loss of CRL4-dependent regulation of CDT1 described in previous reports (see Discussion). The replication licensing factor CDT1 is nearly completely degraded within 24 h after genotoxic stress caused by cisplatin in CRL4-proficient cells (Additional file 1: Figure S4a and S4b). Instead, the CUL4A/B depletion results in a pronounced stabilization of CDT1, such that the cells maintain high CDT1 levels despite cisplatin exposure. This results in uncoupling of the minichromosome maintenance (MCM) helicase activity and an uncontrolled re-replication that triggers RPA recruitment and other ATR-dependent signaling reactions [36]. However, this RPA recruitment to chromatin was not or only slightly further increased by cisplatin treatment of CRL4-deficient cells. As a consequence, CUL4A/B-depleted cells exposed to cisplatin display significantly lower levels of RPA foci when compared to CRL4-proficient controls treated with the same cisplatin concentrations (Fig. 3a and b).

Next, we tested whether RPA recruitment to chromatin in response to cisplatin damage is related to the ssDNA formation. For that purpose, ssDNA induction was assessed using a well-established method based on the incorporation of 5-iodo-2′-deoxyuridine (IdU), which allows for the probing of cells with an antibody that binds to IdU only in the ssDNA conformation. Using this same approach, Huang et al. [5] did not detect ssDNA intermediates after 4- to 6-h treatments with MMC or psoralen (plus UV-A radiation). In agreement with this earlier report, we also observed that ssDNA as well as pS33 remain below the detection threshold within the first 6 h of cisplatin exposure (Additional file 1: Figure S4c-e). However, a longer incubation time of 24 h revealed the formation of clearly detectable ssDNA foci in control cells (Fig. 3a). Again, an increase of ssDNA was observed in CUL4A/B-deficient cells even without genotoxic treatment, as expected from the loss of CRL4-dependent licensing regulation and the notion that the display of ssDNA provides an initial signal for ATR-mediated S phase checkpoint activation upon uncontrolled re-replication [36]. Consistent with the differential recruitment of RPA, ssDNA foci were substantially reduced in CUL4A/B-depleted cells compared to CRL4-proficient controls exposed to the same cisplatin concentrations (Fig. 3a and c). It is important to ascertain in these experiments that IdU is equally incorporated into DNA under the different experimental conditions, as shown by immunofluorescence after DNA denaturation (Additional file 1: Figure S4f-g).

To demonstrate the general relevance of the above-described findings, we also assessed the appearance of ssDNA and consequent RPA phosphorylation in MMC-exposed HeLa and SKOV3 cells (Fig. 3d and e). Immunofluorescence quantifications confirmed that the CUL4A/B depletion counteracts partially the ICL-dependent display of ssDNA and pS33 upon exposure to the crosslinking agent (Fig. 3f and g). Decreased pS33 and pS4/8 levels upon CUL4A/B depletion were also found in immunoblots following MMC treatment of both HeLa and SKOV3 cells (Additional file 1: Figure S4 h). These results indicate that CRL4 activity is required for the generation of a ssDNA-RPA signaling platform essential for the DDR mitigating the cytotoxicity of crosslinking agents.

### FANCD2-dependent ssDNA formation upon cisplatin treatment

**Fig. 4:**
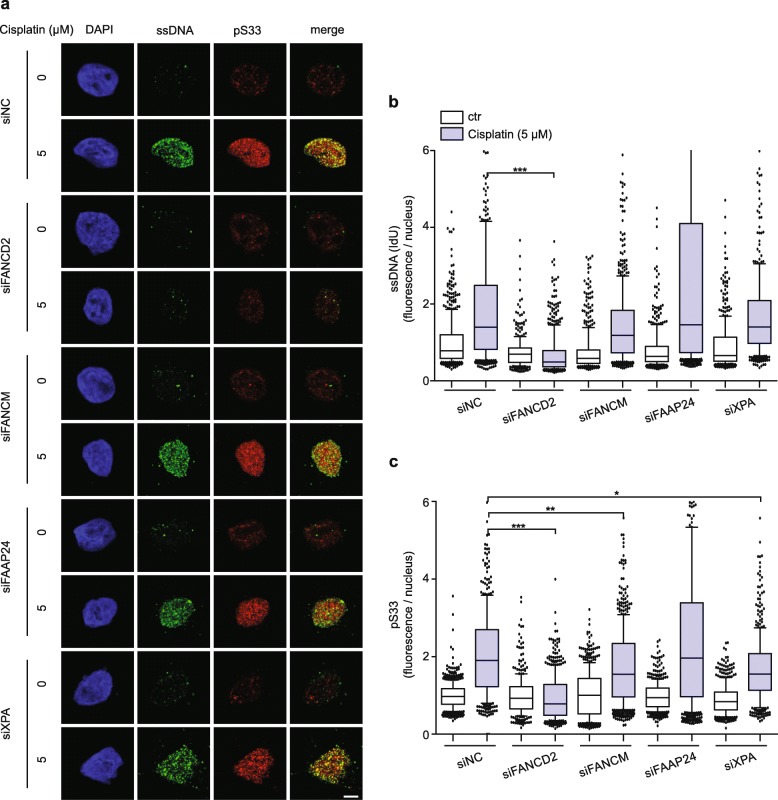
FANCD2-dependent ssDNA formation upon cisplatin treatment. **a** HeLa cells were transfected with siRNA and labeled with IdU prior to cisplatin exposure for 24 h. After fixation, cells were stained for ssDNA, using an anti-IdU antibody, and for pS33 (RPA2 phosphorylated at Ser33). DAPI was employed to visualize the nuclei. **b** Quantification of nuclear fluorescence representing ssDNA induced by the indicated treatments (*N* = 368–594 nuclei from 3 experiments). **c** Quantification of nuclear fluorescence representing RPA2 phosphorylation at Ser33 (*N* = 440–580 nuclei from 2 to 3 experiments). In panels **b** and **c**, horizontal lines represent median values, boxes 25-75th percentiles and whiskers 10-90th percentiles. *P < 0.05, **P < 0.01, ***P < 0.001 (1-way ANOVA according to Kruskal-Wallis). Scale bar: 10 μm

The FA pathway is responsible for the recruitment of nucleases required for the unhooking of crosslinked bases, thus inducing double strand breaks and ssDNA intermediates at ICL sites [37–42]. To corroborate the role of CRL4 in stimulating the formation of ssDNA-RPA complexes in cisplatin-treated cells, we exploited the increased level of ssDNA foci induced by 5-μM cisplatin incubation for 24 h (Fig. 4a and b). After this incubation treatment for 24 h, 95 ± 4.3% (*N* = 7) of control cells remain viable, arguing against the possibility that the accumulation of ssDNA is due to replication catastrophe caused by severely damaged DNA. We then depleted, by siRNA transfection, different members of the FA pathway that have been implicated in DNA damage processing and RPA recruitment. Immunofluorescence analyses (Fig. 4a) and subsequent quantifications (Fig. 4b) revealed that a depletion of FANCD2 is sufficient to prevent ssDNA formation detected after 24 h of cisplatin treatment. This observation, although unexpected in view of previous findings focusing on 4–6 h as the time points for analyses [5], is in line with the notion that FANCD2 constitutes a central member of the FA pathway that organizes downstream effector nucleases [37–40]. This dependence on FANCD2 indicates that ssDNA formation is triggered by ICLs rather than other forms of damage resulting from cisplatin. We also used the siRNA-mediated strategy to down regulate the upstream FA pathway members FANCM and FAAP24 [43, 44] as well as the core nucleotide excision repair subunit XPA. Unlike FANCD2, depletion of these factors failed to detectably reduce ssDNA formation during the same 5-μM cisplatin treatment for 24 h (Fig. 4a and b). It is possible, however, that low residual level of these factors remaining after siRNA transfections (see Additional file 1: Figure S2 for the efficiency of the siRNA-mediated depletion) were sufficient for their action in the display of ssDNA after ICL induction.

Additionally, RPA2 phosphorylation on serine 33 was assessed (Fig. 4a and c) to prove that depletion of FANCD2 not only suppresses the formation of ssDNA but also the consequent foci of phosphorylated RPA2 in cisplatin-exposed cells. This result was confirmed by immunoblots using two distinct siRNA sequences to deplete FANCD2 (Additional file 1: Figure S5a and S5b). In all cases, the lack of FANCD2 reduced markedly the level of pS33 in cisplatin-treated cells. We concluded that the experimental conditions of our study verify the involvement of FANCD2 in the induction of ssDNA serving as a hub for the initiation of RPA signaling at ICL sites.

### CUL4A/B depletion impedes recruitment of FANCD2 and XPF-ERCC1

**Fig. 5:**
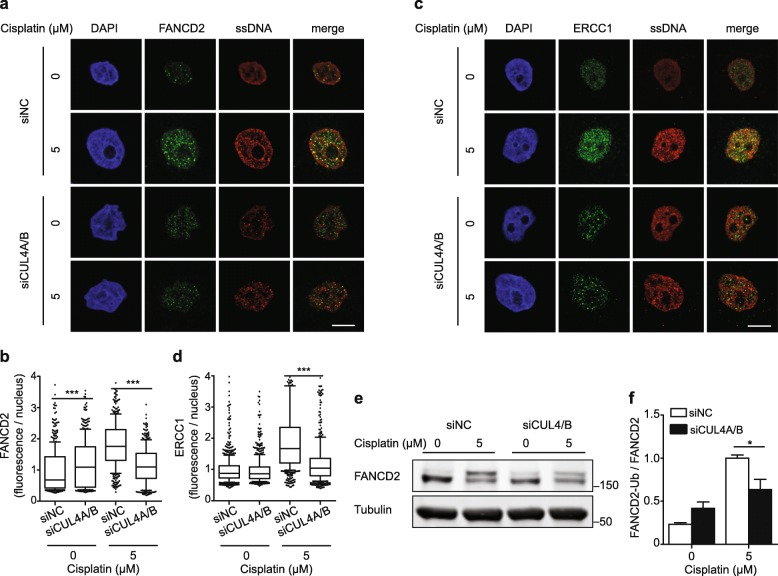
CRL4 depletion impairs recruitment of FANCD2 and XPF-ERCC1. **a** HeLa cells were transfected with siRNA and labeled with IdU prior to 24-h cisplatin exposures. After fixation, cells were stained for FANCD2 and ssDNA (using an anti-IdU antibody). DAPI was used to visualize the nuclei. **b** Quantification of nuclear fluorescence representing FANCD2 foci induced as indicated (*N* = 590–700 nuclei from 3 experiments). **c** Cells were transfected with siRNA and labeled with IdU prior to 24-h cisplatin exposures. After fixation, cells were stained for ERCC1 and ssDNA. DAPI was used to visualize the nuclei. **d** Quantification of nuclear fluorescence representing ERCC1 foci (*N* = 350–480 nuclei from 3 experiments). **e** Immunoblot showing the monoubiquitination of FANCD2 after 24-h cisplatin exposures. Upper bands represent monoubiquitinated FANCD2. Tubulin was used as the loading control. **f** Ratio of monoubiquitinated FANCD2 (FANCD2-Ub) and unmodified FANCD2 (N = 3). In panels b and d, horizontal lines represent median values, boxes the 25-75th percentiles and whiskers the 10-90th percentiles. ***P < 0.001 (1-way ANOVA according to Kruskal-Wallis). In panel **f**, data are presented as mean ± SEM. *P ≤ 0.05 (unpaired, two-tailed t-test). Scale bar: 10 μm

The above results prompted us to use FANCD2 as the molecular target to test whether CRL4 might impact on the FA pathway in cisplatin-damaged cells. A central step in repair of ICLs is monoubiquitiation of FANCD2 by the FA core machinery. Subsequently, a nuclease complex that includes the structure-specific endonuclease XPF-ERCC1 is recruited to chromatin in order to unhook the ICLs [30, 38, 45]. As expected, exposure to cisplatin increases the nuclear foci of FANCD2 and ERCC1, and, interestingly, this recruitment is stimulated by CRL4 activity. The down regulation of CUL4A/B reduces the level of FANCD2 foci (Fig. 5a and b) as well as the level of ERCC1 foci in cisplatin-treated cells (Fig. 5c and d). These findings imply that XPF-ERCC1 may play a key role in the formation of ssDNA at ICLs. This view is supported by the observation that down regulation of ERCC1 with two different siRNA sequences reduces both the ssDNA foci and the consequent RPA phosphorylation in cisplatin-treated cells (Additional file 1: Figure S5c-e).

As stated above, monoubiquitination of FANCD2 is a key prerequisite for recruitment of the downstream nuclease complex including XPF-ERCC1 to ICL sites [37]. We therefore tested whether CRL4 might influence the FANCD2 ubiquitination in response to crosslinking agents. Upon analysis in immunoblots, the monoubiquitination of FANCD2 is indeed impaired in CUL4A/B-depleted cells relative to CRL4-proficient controls (Fig. 5e). Quantifications are presented in Fig. 5f as the ratio of ubiquitinated and unmodified FANCD2 for each condition over three independent experiments. After treatment with 5-μM cisplatin, ~ 50% of FANCD2 is ubiquitinated in control cells. Instead, in CUL4A/B-depleted cells, only ~ 30% of FANCD2 molecules appear in this modified form after the same cisplatin exposure. These results indicate that CRL4 activity stimulates the monoubiquitination of FANCD2 and, accordingly, the FANCD2-dependent recruitment of downstream nucleases.

### CUL4A/B depletion suppresses the S phase checkpoint after exposure to crosslinking agents

**Fig. 6:**
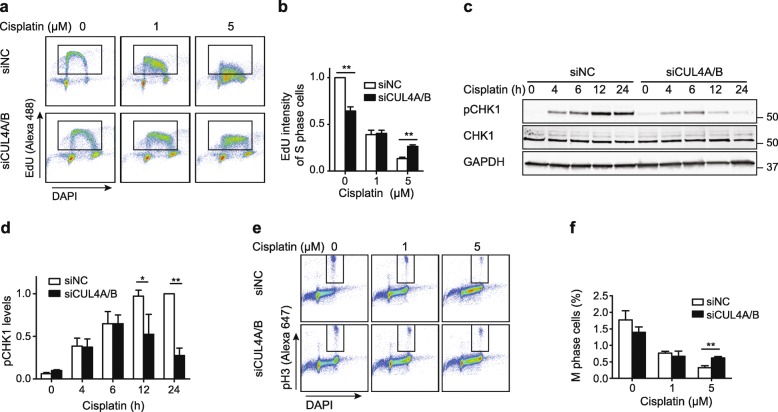
Suppression of the S phase checkpoint upon CRL4 depletion. **a** HeLa cells were siRNA-transfected and incubated for 24 h with cisplatin. The resulting cell cycle distribution was analyzed by flow cytometry. Rectangles contain S phase cells. **b** Quantification of EdU incorporation in S phase cells after the indicated treatments. EdU intensities are normalized to siNC controls not exposed to cisplatin (N = 5). **c** Immunoblot showing pCHK1 (phosphorylated at Ser345) after exposure to 20 μM cisplatin. **d** Quantification of pCHK1 normalized to GAPDH. Values are expressed relative to the amount of pCHK1 in CRL4-proficient cells treated for 24 h with cisplatin (N = 3–5). **e** CUL4A/B-depleted cells were incubated for 24 h with cisplatin as indicated. Mitotic cells were stained with antibodies against pH3 and DAPI, and analyzed by flow cytometry. Rectangles indicate pH3-positive cells. **f** Percentage of mitotic cells resulting from the indicated treatments (N = 3–5). In panels **b**, **d**, **f**, data are presented as mean ± SEM. *P ≤ 0.05, **P < 0.01 (unpaired, two-tailed t-test)

Upon DNA damage, replication is inhibited by S phase checkpoints to ensure repair of the damage before cells enter mitosis, which is an important strategy to prevent cell death by replication catastrophe. As the above described results indicate that CRL4 supports damage signaling by stimulating the FANCD2-ERCC1-dependent formation of the ssDNA-RPA complexes, we next assessed whether the abrogated signaling in CRL4-deficient cells affects S-phase progression. HeLa cells depleted of CUL4A/B were incubated for 24 h with cisplatin or MMC. Thereafter, DNA content and DNA synthesis were monitored by measuring 4′,6-diamidino-2-phenylindole (DAPI) binding and 5-ethynyl-2′-deoxyuridine (EdU) incorporation, respectively, in flow cytometry analyses. When cells were transfected with non-coding control RNA, cisplatin inhibited their replicative DNA synthesis in a dose-dependent manner (Fig. 6a and b). At a cisplatin concentration of 5 μM, the EdU incorporation was decreased by nearly 90%. As expected [20], the CUL4A/B depletion on its own was sufficient to elicit intra-S phase checkpoint responses lowering the rate of DNA synthesis. However, such a reduced DNA synthesis compared to non-coding siRNA controls was observed only as long as the cells were not exposed to cisplatin. This is demonstrated by the fact that the combination of CUL4A/B depletion and 5-μM cisplatin treatment resulted in a 2-fold higher EdU incorporation compared to the same cisplatin treatment in CRL4-proficient controls (Fig. 6b).

This elevated DNA synthesis is in agreement with the dampened activation of the intra-S checkpoint transducer CHK1, a direct target of ATR. An increase of CHK1 phosphorylation (generating pCHK1) was observed in CRL4-proficient HeLa cells treated with cisplatin lasting at least 24 h after initiation of drug exposure (Fig. 6c and d). Although there was an initial increase of pCHK1 in CRL4-deficient cells, at later timepoints pCHK1 levels were significantly lower. In MMC-exposed cells, CUL4A/B depletion also impedes CHK1 phosphorylation, albeit partially (Additional file 1: Figure S6a). Although CHK1 had been identified as a possible CRL4 substrate [46], we did not observe any overall changes of CHK1 protein level. One may argue that the reduced phosphorylation of CHK1 in CUL4A/B-depleted cells exposed to crosslinking agents results from a compromised viability. However, over three independent experiments the combined CUL4A/B deficiency had no statistically significant influence on the phosphorylation of CHK2 protein in cisplatin-treated cells (Additional file 1: Figure S6b and S6c). These findings indicate that CRL4 activity stimulates mainly the ATR/CHK1 signaling pathway in cells treated with crosslinking agents.

An identical response with stimulation of DNA synthesis in CRL4-deficient relative to CRL4-proficient counterparts was detected after exposure to MMC (Additional file 1: Figure S6d and S6e). These findings confirm that the cells respond to cisplatin and MMC treatment with an effective S phase checkpoint that suppresses DNA synthesis. However, this cell cycle checkpoint is at least in part abrogated by concomitant depletion of the CRL4 scaffold proteins CUL4A and CUL4B, such that cisplatin- or MMC-exposed and CUL4A/B-depleted cells display higher rates of DNA synthesis than control cells treated with these same crosslinking drug. Cell cycle analyses established that a depletion of FANCD2 or ERCC1 results in a defective S phase checkpoint and elevated DNA synthesis in cisplatin-treated cells (Additional file 1: Figure S6f and S6 g) exactly as observed after CUL4A/B depletion (Fig. 6a). This identical checkpoint defect is consistent with the notion that CRL4 activity positively regulates the observed ICL –> FANCD2 –> XPF-ERCC1 –> ssDNA-RPA –> ATR/CHK1 pathway.

We next tested whether the weakened S phase checkpoint, translating to an accelerated S phase progression, leads to an increase of the M phase population. For that purpose, cisplatin-treated cells were stained for DNA content and histone H3 phosphorylation at position Ser10 (pH3), a well-established marker of mitosis, and subsequently analyzed by flow cytometry (Fig. 6e). The proportion of cells reaching M phase was reduced upon cisplatin exposure in a dose-dependent manner. However, the CUL4A/B depletion doubled the fraction of M phase cells relative to the non-coding siRNA controls (Fig. 6f). Because the tested cisplatin concentration of 5 μM is toxic in CUL4A/B-depleted cells, we concluded that the CUL4A/B deficiency allows for entering mitosis despite irreparable DNA damage, thereby causing mitotic catastrophe.

### Inhibition of Neddylation recapitulates the effects of CUL4A/B depletion

**Fig. 7:**
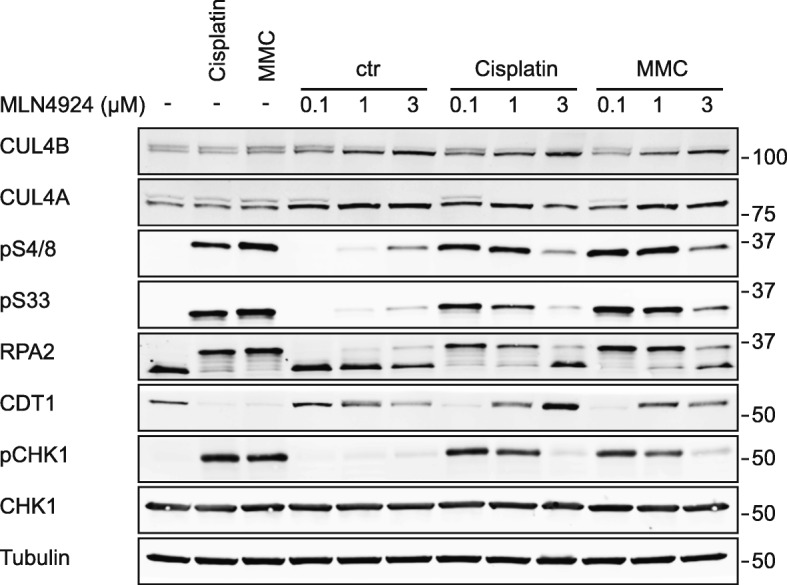
MLN4924 recapitulates the effects of CUL4A/B depletion. HeLa cells were treated simultaneously with an ICL-inducing agent and MLN4924 as indicated. The cisplatin and MMC concentrations were 5 μM and 1 μM, respectively. After 24 h, cells were analyzed to determine CUL4A/B modifications, RPA2, CDT1 and CHK1, and the phosphorylation of RPA2 (pS4/8 and pS33) and CHK1 (pCHK1). Tubulin served as the loading control

Our results suggest that the cytotoxic effect of the neddylation inhibitor MLN4924 is mediated partially by inactivation of the CRL4 ligase (Fig. 1). For further confirmation, we examined whether MLN4924 would reiterate the effect of CUL4A/B depletion on the S-phase checkpoint regulation following cisplatin and MMC exposure (Fig. 7). Efficient CRL4 inhibition is indicated by the disappearance of the slower migrating CUL4 bands, representing the active neddylated form, upon incubation of HeLa cells with MLN4924. The inhibitor alone only weakly activates the DNA damage response, reflected by slight increases in pS33, pS4/8 and pCHK1, whereas cisplatin and MMC induce a pronounced phosphorylation of RPA2 and CHK1, accompanied by nearly complete degradation of CDT1. This ICL-induced DNA damage response was progressively suppressed in the presence of increasing MLN4924 concentrations, thus supporting the described role of CRL4 in stimulating FA pathway-induced ssDNA signaling.

## Discussion

This study was instigated by the surprising observation that, in the presence of the DNA-crosslinking agents cisplatin and MMC, a CRL4 deficiency causes the same cell cycle checkpoint defect as a depletion of FANCD2 or ERCC1. Therefore, our findings indicate that CRL4 positively regulates a FANCD2- and ERCC1-dependent checkpoint response that depends on the local deployment of ssDNA. This involvement of the FA pathway, which is dedicated to the repair of ICLs [8, 31, 32], implies that the ICL lesions induced by cisplatin and MMC constitute the actual trigger for the observed ssDNA induction. Cisplatin, MMC and other crosslinking agents are highly cytotoxic because the resulting ICLs interfere with processes requiring separation of the two DNA strands and thereby block DNA replication [1]. Although it is well known that the repair of ICLs during DNA replication requires the FA pathway, the mechanism by which crosslinking agents induce the S phase checkpoint is less well understood. A previous study, describing the role of the FA pathway after 4- to 6-h treatments with MMC or psoralen (plus ultraviolet irradiation), did not detect any ssDNA intermediates. The authors of that earlier study reported that, during this time window of 4–6 h, the FA pathway members FANCM and FAAP24 are able to initiate an RPA-dependent checkpoint response to ICLs without generating ssDNA intermediates [5]. In agreement with this earlier report, we also observed that ssDNA levels remain below the detection threshold within 6 h of cisplatin or MMC exposure. However, abundant ssDNA foci, which depend on FANCD2 for the recruitment of a nuclease complex comprising ERCC1, are detected after 24-h treatments with these same crosslinking agents. The ssDNA foci emerging in a time-dependent manner ultimately reinforce an S phase checkpoint by recruitment of RPA and subsequent engagement and activation of the ATR and CHK1 protein kinases. We conclude that, in addition to the previously discovered ssDNA-independent and short-term checkpoint response to ICLs, there is a ssDNA-dependent and sustained response to the same lesions, together culminating in cell cycle arrest. We unexpectedly observed that cells lacking CLR4 activity are impaired in this ssDNA-dependent signaling response to ICLs and the functional consequence of our finding is that a CLR4 deficiency potentiates the cytotoxicity of cisplatin and MMC.

Our report establishes an unforeseen functional link between two distinct ubiquitination systems, i.e., the FA pathway and CRL4 complexes. CRL4 ubiquitin ligases are formed by assembly of one of two closely related scaffold proteins (CUL4A or CUL4B) with the adaptor protein DDB1, which associates with substrate receptors. The scaffold subunit also binds to the RING finger protein RBX1 mediating the association with ubiquitin-delivering enzymes. The two paralogs CUL4A and CUL4B share high sequence similarity. Importantly, these CUL4A/B paralogs are amplified or overexpressed in human carcinomas and provide negative prognostic markers for survival [47]. CRL4 complexes employ a variety of substrate receptors that target specific proteins for ubiquitination [12, 13]. For example, the CRL4^CDT2^ ubiquitin ligase promotes degradation of the replication licensing factor CDT1 after replication origin firing to ensure that DNA is replicated only once per cell cycle [18, 48]. Exposure to DNA-damaging agents also induces rapid CDT1 proteolysis through CRL4-mediated ubiquitination [49, 50], whereas ectopic CDT1 expression promotes DNA re-replication [18, 20]. Accordingly, CRL4-deficient cells display higher constitutive levels of CDT1 and the resulting deleterious re-replication has been shown to activate DDR signaling [20, 35, 36]. A cursory interpretation of our findings might, therefore, implicate the aberrant stabilization of cell cycle factors like CDT1 and p21, as described to explain the efficacy of pevonedistat against melanoma cells [51], together with deregulated MCM activity [36], as the cause of an increased sensitivity of CRL4-deficient cells to crosslinking agents. However, the lack of CRL4 increases ssDNA levels only in undamaged cells and this slightly higher ssDNA content, seen in comparison to CRL4-proficient counterparts, is not further enhanced by exposure to cisplatin or MMC. On the contrary, in the presence of such crosslinking agents the extent of ssDNA remains significantly lower in CRL4-deficient cells compared to the CRL4-proficient controls. This observation points to an additional, fundamentally different effect of CRL4 down regulation that suppresses the ICL-induced ssDNA formation, thus limiting ssDNA-RPA signaling and the consequent activation of checkpoint kinases. Consequently, the ICL-triggered phosphorylation of RPA2 (at position Ser33) and the phosphorylation of downstream effectors like H2AX is reduced in cells lacking CRL4 activity compared to CRL4-proficient controls.

**Fig. 8:**
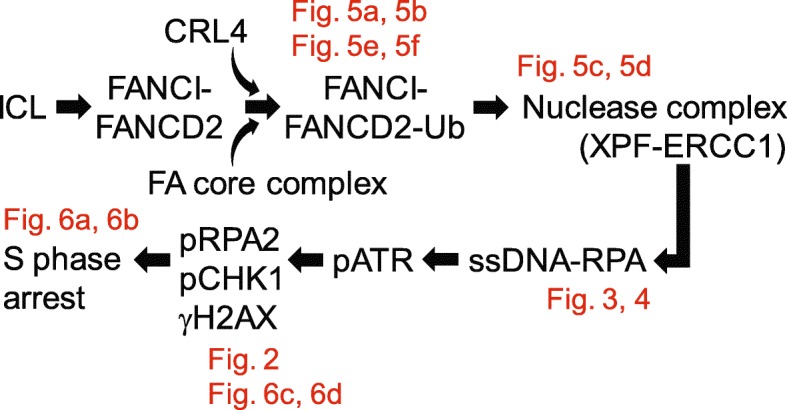
Scheme describing the function of CRL4 in protecting cancer cells from ICL cytotoxicity. The results of Figs. 3, 4, 5 and 6 indicate that CRL4 stimulates the FA pathway-dependent ubiquitination of the FANCI-FANCD2 complex, which is necessary for the recruitment of nucleases to ICL sites [8, 31, 32]. In turn, nuclease activity at ICLs generates a ssDNA-RNA platform for activation of an ATR-dependent cell cycle checkpoint signaling leading to S phase arrest. This findings imply that CRL4 provides a potential target for the sensitization of cancer cells to crosslinking drugs

A possible mechanistic basis for the ability of CRL4 to support an S phase checkpoint in response to crosslinking agents is provided by the finding that ssDNA formation at ICLs is dependent on robust monoubiquitination of the FA pathway member FANCD2. Once monoubiquitinated, this factor is known to recruit nucleases including XPF-ERCC1 to ICL sites [8, 37–40, 52]. We observed that CRL4 activity supports the monoubiquitination of FANCD2 and that this enhanced modification is needed for the nuclease recruitment to ICLs (Fig. 8). XPF-ERCC1 is a structure-specific endonuclease that unhooks the ICLs without yielding large stretches of ssDNA. However, subsequent DNA resection for example by the MRE11-RAD50-NBS1 complex or by CtIP, which also interact with FANCD2 [41, 53], may ultimately be responsible for the observed ssDNA foci at ICLs. In any case, our findings suggest that the fraction of monoubiquitinated FANCD2 must exceed a critical threshold to trigger the recruitment of XPF-ERCC1 to an extent that promotes the display of ssDNA in response to ICLs. It is not clear whether FANCD2 is a direct target of ubiquitination by CRL4 or whether CUL4A/B depletion affects other members of the FA pathway supporting the ubiquitination of FANCD2. Nonetheless, our findings indicate that CRL4 activity is needed to overcome this critical threshold, such that down regulation of CRL4 reduces the formation of ssDNA at ICLs. The relevance of these findings is broadened by the observation that CLR4 activity is needed for the S phase checkpoint in response to both cisplatin and MMC treatment, and that this role of CLR4 in ICL-dependent checkpoint signaling extends to SKOV3 ovarian carcinoma cells.

## Conclusion

Taken together, at least a subset of cancer cells is vulnerable to the combination of a crosslinking agent and CRL4 inhibition. One future challenge is to develop selective CRL4 inhibitors to avoid side effects due to the unnecessary blockage of other cullin-type ubiquitin ligases. Also, it is necessary to discover and validate biomarkers for the identification of those cancer subsets that are most susceptible to a combined treatment of crosslinking agents and CRL4 inhibitors. Our study provides insight into a novel function of CRL4 in mitigating the cytotoxicity of ICLs through stimulation an FA pathway-dependent checkpoint response. Consequently, CRL4 is a potential new therapeutic target to improve the anticancer efficacy of ICL-inducing drugs.

## Supplementary information

**Additional file 1: Figure S1.** CRL4 inhibition potentiates ICL cytotoxicity **Figure S2.** Depletion efficiency after siRNA transfections. **Figure S3.** Impaired RPA phosphorylation in CRL4-deficient cells. **Figure S4.** CRL4 dependent assembling of ssDNA-RPA complex. **Figure S5.** FANCD2 depletion impairs RPA2 phosphorylation. **Figure S6.** CRL4 supports the S-phase checkpoint response. **Table S1**. Oligonucleotide sequences **Table S2.** Antibodies.

## Data Availability

All data generated or analyzed during this study are reflected in the present published article and its supplementary information files.

## Abbreviations

ATR: Ataxia telangiectasia-mutated and Rad3-related
CHK1: Checkpoint kinase 1
CRL: Cullin-RING ubiquitin ligases
DAPI: 4′,6-Diamidino-2-phenylindole
DDB1: Damaged DNA-binding 1
DDR: DNA damage response
EdU: 5-Ethynyl-2′-deoxyuridine
FA: Fanconi anemia
ICL: Interstrand crosslink
IdU: 5-Iodo-2′-deoxyuridine
MCM: Minichromosome maintenance
MMC: Mitomycin C
RPA: Replication protein A
ssDNA: Single-stranded DNA
