# Supplementary material for: CRL4 ubiquitin ligase stimulates Fanconi anemia pathway-induced single-stranded DNA-RPA signaling
Source: BMC Cancer. 2019 Nov 5;19:1042. doi: 10.1186/s12885-019-6305-x (PMC6833152; doi:10.1186/s12885-019-6305-x)
Supplement: Supplementary file 1 — Additional file 1: Figure S1. CRL4 inhibition potentiates ICL cytotoxicity Figure S2. Depletion efficiency after siRNA transfections. Figure S3. Impaired RPA phosphorylation in CRL4-deficient cells. Figure S4. CRL4 dependent assembling of ssDNA-RPA complex. Figure S5. FANCD2 depletion impairs RPA2 phosphorylation. Figure S6. CRL4 supports the S-phase checkpoint response. Table S1. Oligonucleotide sequences Table S2. Antibodies. [file 12885_2019_6305_MOESM1_ESM.docx]

**Additional file 1: Supplementary figures and tables**


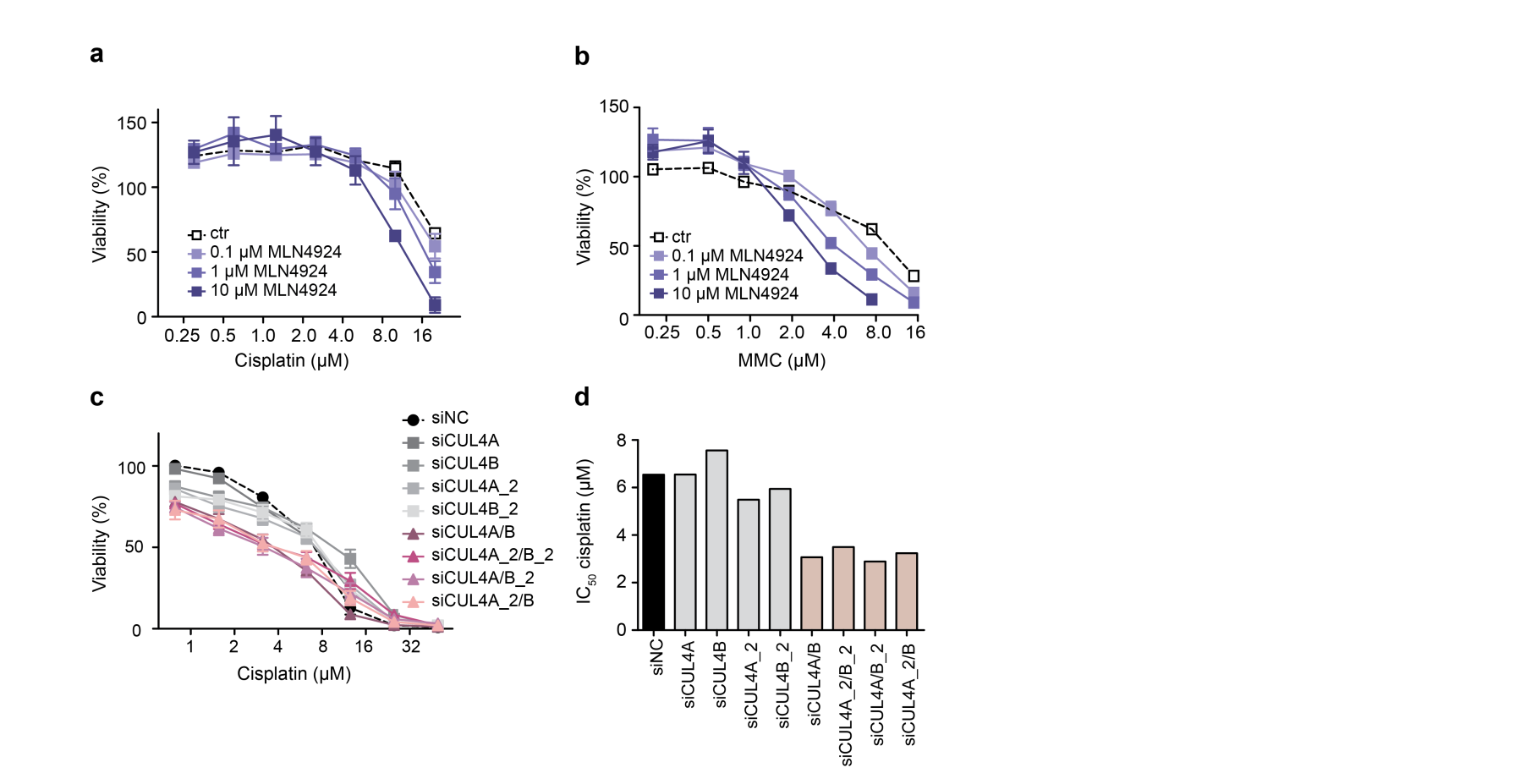


**Figure S1.** CRL4 inhibition potentiates ICL cytotoxicity. **a** SKOV3 cells were incubated for 48 h with cisplatin in combination with MLN4924 as indicated. Cell viability is expressed as the percentage of control values obtained in the absence of cisplatin (N = 3 independent experiments, error bars show s.e.m.). **b** SKOV3 cells were incubated for 48 h with MMC in combination with MLN4924 as indicated. Cell viability is expressed as the percentage of control values obtained in the absence of MMC (N = 3). **c** Two different siRNAs for CUL4A and CUL4B were used either alone or in combination to down regulate the CRL4 complex in HeLa cells. Transfected cells were incubated with the indicated concentrations of cisplatin and their viability was tested after 48 h (N = 3). **d** IC_50_ values of cisplatin calculated from panel **c**.


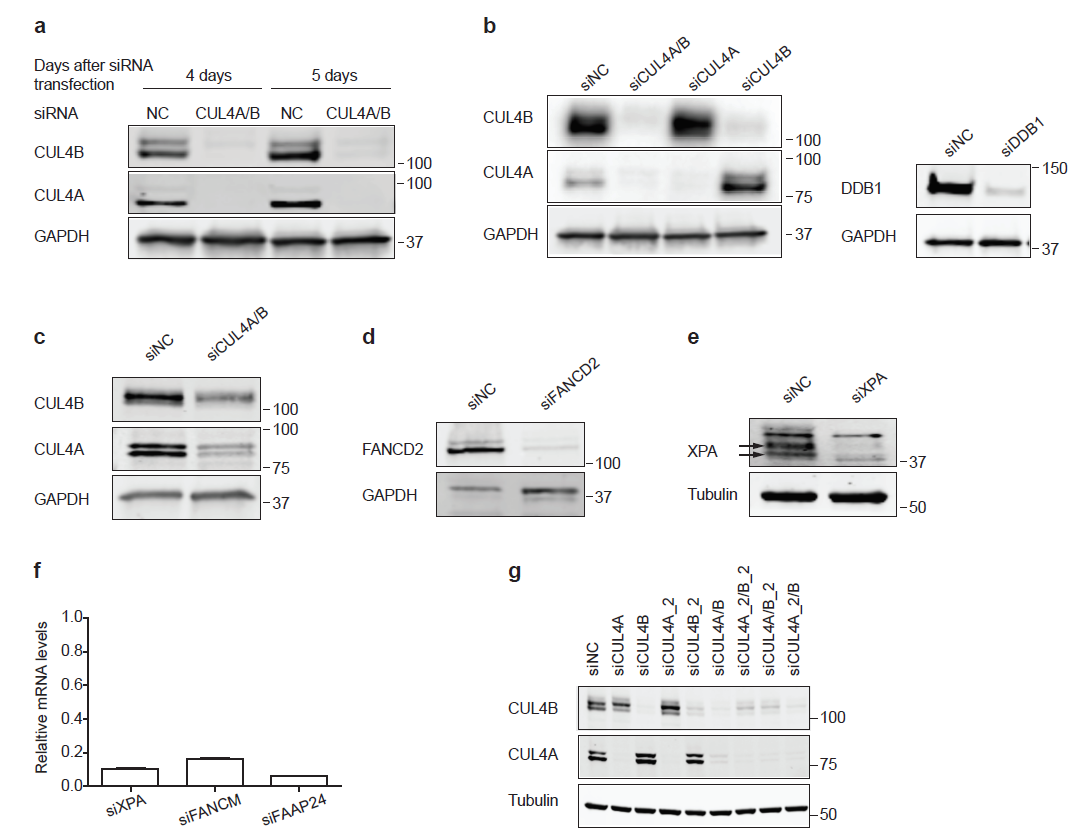


**Figure S2.** Depletion efficiency after siRNA transfections. **a** Immunoblots demonstrating the down regulation of CUL4A and CUL4B proteins in HeLa cells at the indicated times after siRNA transfections. GAPDH was used as the loading control. **b** CUL4A/B and DDB1 levels in HeLa cells 3 days after transfection with siRNA reagents. **c** CUL4A/B levels in SKOV3 cells 4 days after transfection with siRNA reagents. **d** FANCD2 levels in HeLa cells 4 days after transfection with siRNA reagents. **e** Levels of XPA (migrating as a double band) in HeLa cells 4 days after transfection with siRNA reagents. **f** Residual XPA, FANCM and FAAP25 mRNA levels (relative to siNC controls) quantified by quantitative RT-PCR 3 days after siRNA transfection of HeLa cells. GAPDH was used as internal standard. Error bars show s.e.m. **g** Depletion of CUL4A/B using additional siRNA sequences 3 days after the indicated siRNA transfections.


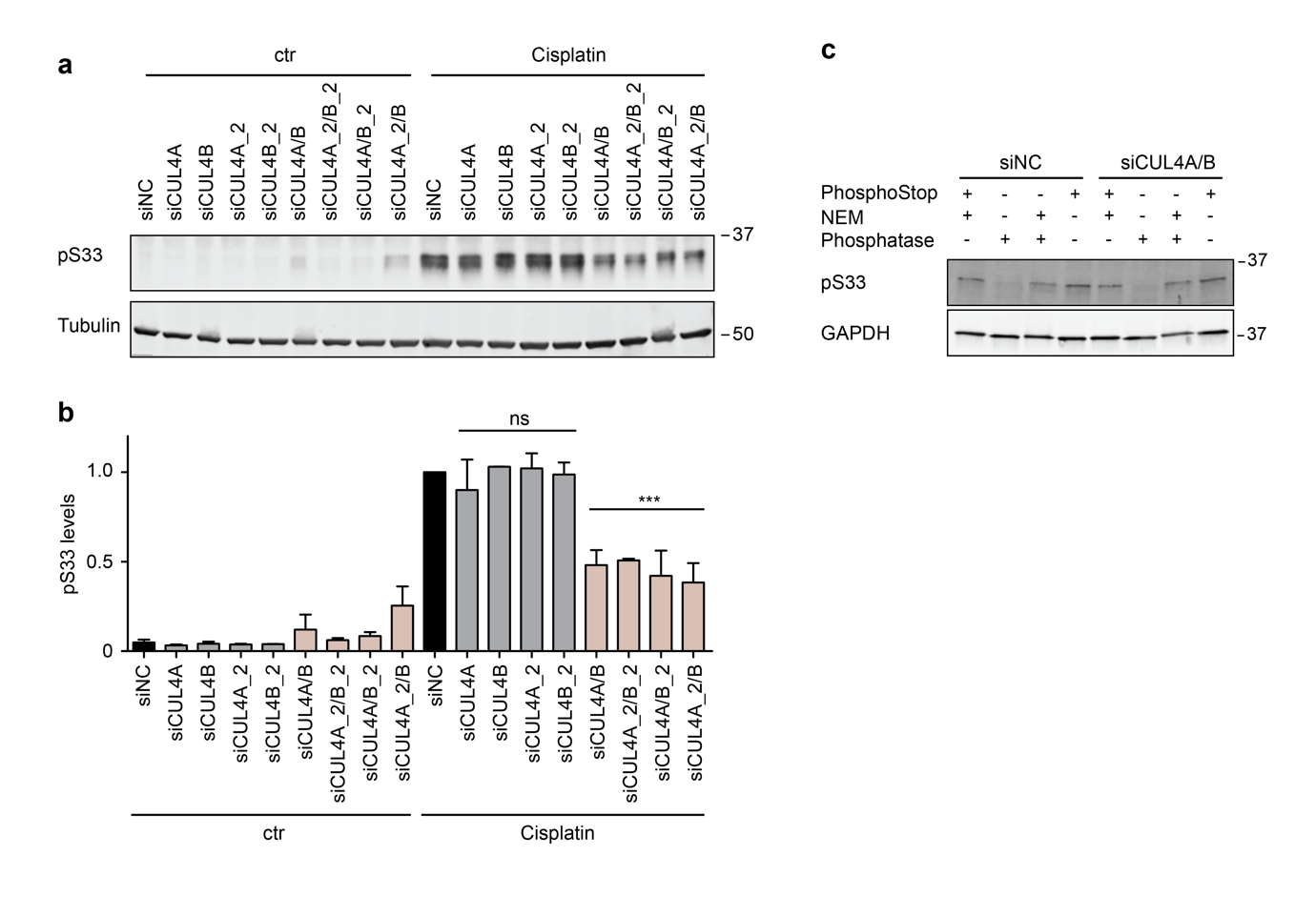


**Figue S3.** Impaired RPA phosphorylation in CRL4-deficient cells. **a** HeLa cells were depleted for CUL4A and CUL4B using two distinct siRNA sequences for each protein, i.e., siCUL4A, siCUL4A_2, siCUL4B and siCUL4B_2, either alone or in combination. Transfected cells were exposed to 5 µM cisplatin for 24 h. Whole cell lysates were probed in immunoblots with the phospho-specific antibody pS33. Tubulin served as the loading control. **b** Quantification of pS33 levels normalized to tubulin. Values are expressed relative to pS33 levels of control cells (siNC) exposed to 5 µM cisplatin (N = 3); asterisks indicate significant lower levels of pS33 in CUL4A/B co-depleted cells relative to non-coding controls, whereas single depleted cells show no significant differences relative to siNC controls (***P < 0.001, unpaired, two-tailed t-test). **c** Control immunoblot demonstrating the susceptibility of pS33 to dephosphorylation by phosphatase treatment. PhosphoStop, phosphatase inhibitor. NEM, N-ethylmaleimide.

**
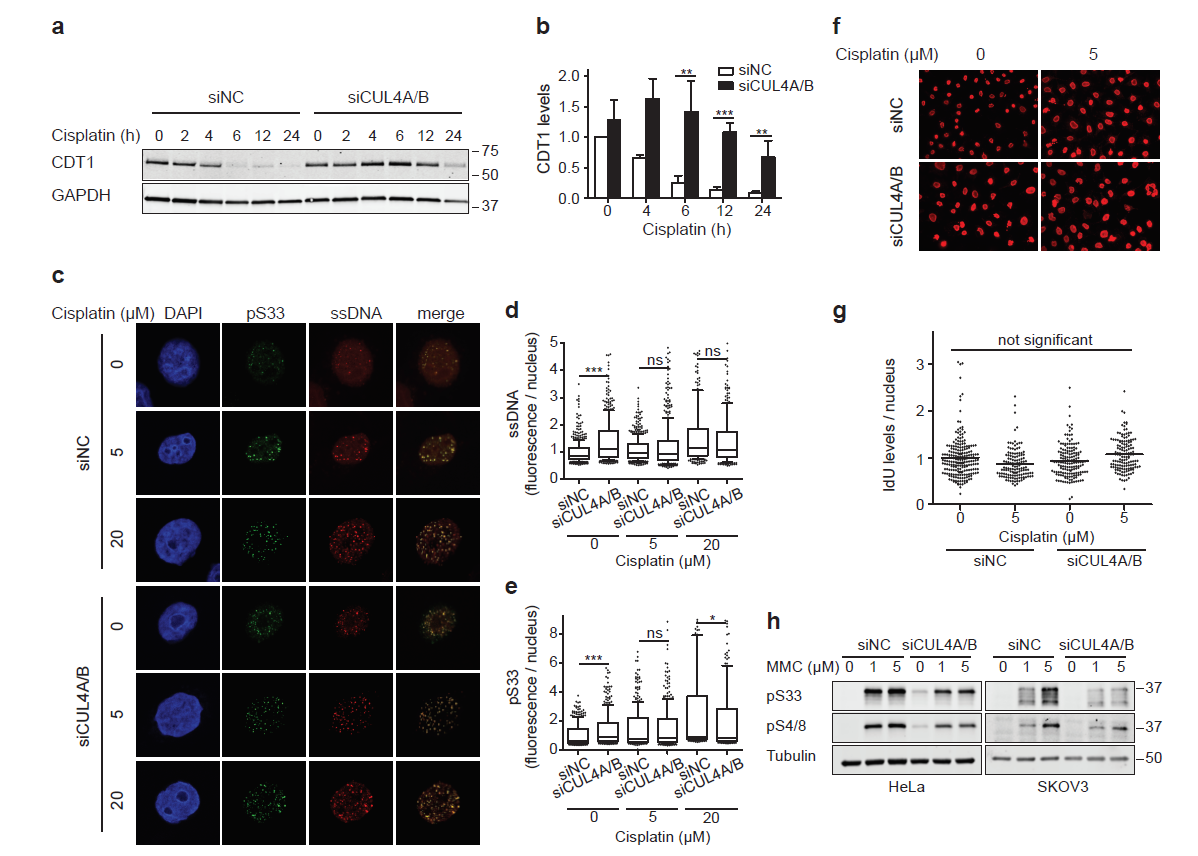
**

**Figure S4.** CRL4 dependent assembling of ssDNA-RPA complex**. a** Representative immunoblot showing CDT1 levels in cells subjected to the indicated siRNA transfections and challenged with 20 µM cisplatin. GAPDH was the loading control. **b** Quantification of CDT1 normalized to GAPDH. Values are expressed relative to the constitutive CDT1 level in untreated cells (N = 3-5). Data are presented as mean ± s.e.m.; **P < 0.01, ***P < 0.001 (unpaired, two-tailed t-test). At the 0 h time point, the observed increase of CDT1 level in CRL4-deficient (siCUL4A/B) compared to CRL4-proficient cells (siNC) was not statistically significant. **c** HeLa cells were transfected with siRNA and labeled with IdU prior to 6-h cisplatin exposures. After fixation, cells were stained for ssDNA, using an anti-IdU antibody, and for pS33. DAPI was used to visualize the nuclei. Quantification of nuclear fluorescence representing **d** ssDNA or **e** pS33 induced as indicated (N = 238-568 nuclei from 2 experiments). **f** Representative images demonstrating that differences in fluorescence intensity are not due to changes in the efficiency of IdU incorporation, as the antibody yielded identical immunofluorescence signals after DNA denaturation, to convert all double-stranded to ssDNA conformations. HeLa cells were transfected with the indicated siRNA sequences and labeled for 30 h with IdU before genotoxic treatment, which consisted of a 24-h exposure to cisplatin. Control cells were mock-treated. For the detection of ssDNA, the cells were fixed and stained with anti-IdU antibodies after denaturation. **g** Quantification of mean nuclear fluorescence obtained after DNA denaturation (N = 200 nuclei). Horizontal lines represent median values. The statistical analysis carried out by 1-way ANOVA according to Kruskal-Wallis revealed no significant differences between treatments. **h** HeLa or SKOV3 cells were transfected with siCUL4A/B, or with siNC, and incubated for 24 h with 1 or 5 µM MMC. Whole cell lysates were probed in immunoblots with phospho-specific antibodies against pS33 and pS4/8. Tubulin served as the loading control.


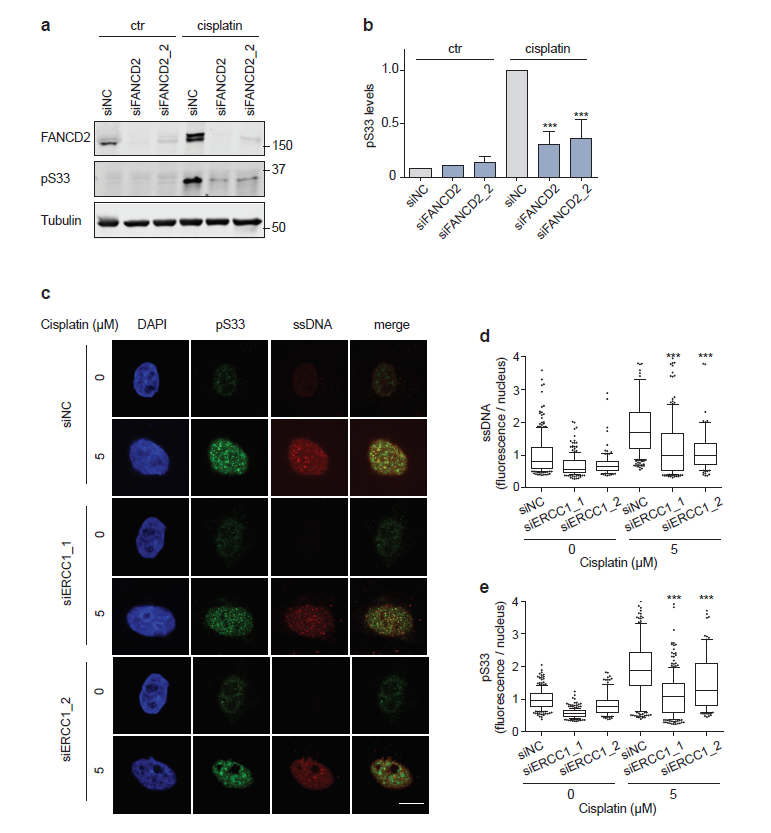


**Fig. S5** FANCD2 depletion impairs RPA2 phosphorylation**. a** FANCD2 was depleted in HeLa cells using two different siRNA sequences, i.e., siFANCD2 and siFANCD2_2 and exposed to 5 µM cisplatin for 24 h. Whole cell lysates were probed in immunoblots with the phospho-specific antibody against pS33. Tubulin served as the loading control. **b** Quantification of pS33 levels normalized to tubulin. Values are expressed relative to cisplatin-exposed control cells (siNC) (N = 3; ***P < 0.001, unpaired two-tailed t-test). **c** HeLa cells were transfected with two different siRNA sequences targeting ERCC1 (siERCC1_1 and siERCC1_2) and labeled with IdU prior to 24-h cisplatin exposures. After fixation, cells were stained for ssDNA, using an anti-IdU antibody, and for pS33. DAPI was used to visualize the nuclei. Quantification of nuclear fluorescence representing **d** ssDNA or **e** pS33 induced as indicated (N = 256-312 nuclei from 2 experiments).


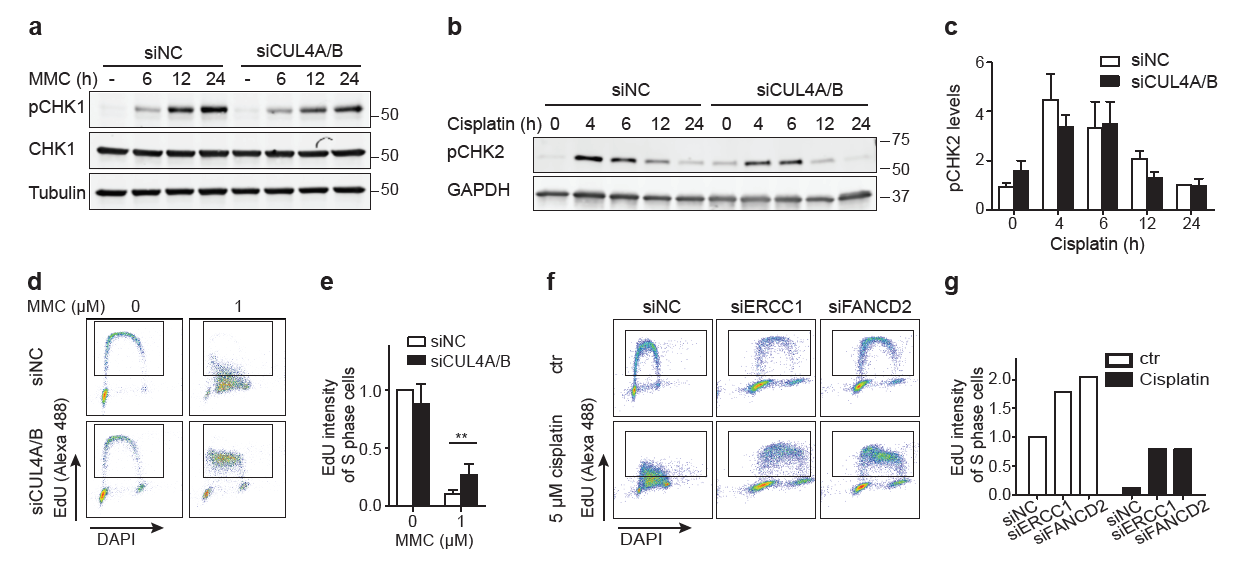


**Fig. S6** CRL4 supports the S-phase checkpoint response. **a** Immunoblot showing pCHK1 induced by 1 µM MMC relative to non-phosphorylated CHK1 and tubulin used as loading standards. **b** Representative immunoblots showing changes of phosphorylated CHK2 (pCHK2) relative to GAPDH used as the loading control. HeLa cells were depleted of CUL4A/B as indicated and incubated with 20 µM cisplatin for different time periods. **c** Quantification of pCHK2 levels normalized to GAPDH cells challenged with 20 µM cisplatin. Values are expressed relative to the pCHK2 levels in the 24 h-treated control cells (N = 3-5); none of the differences were statistically significant. **d** Cells were transfected with siRNA and incubated for 24 h with MMC. The resulting cell cycle distribution was analyzed by flow cytometry. **e** Quantification of EdU intensity in S phase populations normalized to siNC controls not exposed to MMC (N = 5). **f** HeLa cells were siRNA-transfected, incubated for 24 h with cisplatin and the resulting cell cycle distribution was analyzed by flow cytometry. Rectangles contain S phase cells. **g** Quantification of EdU incorporation in S phase cells after the indicated treatments. EdU intensities are normalized to siNC controls not exposed to cisplatin.

**Table S1**: Oligonucleotide sequences

| **siRNAs** |  |  |
| --- | --- | --- |
| **OLIGONUCLEOTIDES** | **SOURCE** | **IDENTIFIER** |
| siCUL4A: UUCGAAGGACAUCAUGGUUCA | Qiagen | SI04245465 |
| siCUL4A_2: AGCGATCGTAATCAATCCTGA | Microsynth | N/A |
| siCUL4B: CACCGUCUCUAGCUUUCUAA | Qiagen | SI03162215 |
| siCUL4B_2: TTGGAGCCGTTAGGAAGATTA | Microsynth | N/A |
| siDDB1: UUUAUUAUCGCGAUCUAGUGG | Qiagen | SI02663402 |
| siFANCD2: UUGGAGGAGAUUGAUGGUCUA | Microsynth | (Vinciguerra et al., 2010) |
| siFANCD2_2: CGGCTTCTCGGAAGTAATTTA | Microsynth | (Huang et al., 2010) |
| siFANCM: AAGCUCAUAAAGCUCUCGGAA | Microsynth | (Vinciguerra et al., 2010) |
| siFAAP24: CCGGAUGAGUGAACAAUACUU | Microsynth | (Huang et al., 2010) |
| siXPA: GCUACUGGAGGCAUGGCUA | Microsynth | N/A |
|  |  |  |
| non-coding siRNA (siNC): AAUUCUCCGAACGUGUCACGU | Microsynth | N/A |
| siCUL1: AACGTAGTTATCAGCGATTCA | Qiagen | SI02225657 |
| siCUL2: CGGCACAATGCCCTTATTCAA | Qiagen | SI02225671 |
| siCUL3: AACAACTTTCTTCAAACGCTA | Qiagen | SI02225685 |
| siCUL5: TACGAGCAGTAAACTTGCCAA | Qiagen | SI05119989 |
| siCUL7: AACCCAAGAGUUUGAUUAUAAAA | Microsynth | N/A |
| siCUL9: AACCACAUCCUCUGAAGAACACU | Microsynth | N/A |
|  |  |  |
| **Primers** |  |  |
| **OLIGONUCLEOTIDES** | **SOURCE** | **IDENTIFIER** |
| FANCM forward ACCAGGTAGTGATATAAAGGCTGT | Microsynth | N/A |
| FANCM reverse CTCTCCCTCGATTATGCCTTGT | Microsynth | N/A |
| FANCD2 forward TTCCGAGAGCTGGACATTGA | Microsynth | N/A |
| FANCD2 reverse TGGCAATAGGAGGTGTCAGC | Microsynth | N/A |
| XPA forward GGCGAGTATCGAGCGGAAG | Microsynth | N/A |
| XPA reverse TGAAGCCTCCTCCTGTGTCA | Microsynth | N/A |
| FAAP24 forward GGATGGCTTGACACCAGACT | Microsynth | N/A |
| FAAP24 reverse ACTGGGCTCTTTGGTTTGCT | Microsynth | N/A |

**Table S2:** Antibodies

| **ANTIBODIES** | **SOURCE** | **IDENTIFIER** | **DILUTIONS** | | |
| --- | --- | --- | --- | --- | --- |
|  |  |  | **WB** | **IF** | **FC** |
| Mouse monoclonal anti-alpha-Tubulin (clone B-5-1-2) | Sigma-Aldrich | Cat# T5168; RRID: AB_477579 | 1:10'000 |  |  |
| Rabbit monoclonal anti-ATM (phospho S1981) (clone EP1890Y) | Abcam | Cat# ab81292; RRID:AB_1640207 | 1:10,000 |  |  |
| Rabbit polyclonal anti-ATR (phospho S428) | Santa Cruz Biotechnology | Cat# sc-109912; RRID:AB_2063314 | 1:100 |  |  |
| Mouse purified anti-BrdU (clone B44) | BD Biosciences | Cat# 347580, RRID:AB_400326 |  | 1:200 |  |
| Rabbit monoclonal anti-CDT1 (clone D10F11) | Cell Signaling Technology | Cat# 8064S; RRID:AB_10896851 | 1:1000 |  |  |
| Mouse monoclonal anti-CHK1 (clone D-7) | Santa Cruz Biotechnology | Cat# sc-377231; RRID:N/A | 1:200 |  |  |
| Rabbit monoclonal anti-CHK1 (phospho S345) (clone 133D3) | Cell Signaling Technology | Cat# 2348; RRID:AB_331212 | 1:1000 |  |  |
| Rabbit polyclonal anti-CHK2 (phospho Thr68) | Cell Signaling Technology | Cat# 2661; RRID:AB_331479 | 1:1000 |  |  |
| Rabbit polyclonal anti-Cullin 4A | Thermo Fisher Scientific | Cat# PA5-17101; RRID:AB_10978708 | 1:1000 |  |  |
| Rabbit polyclonal anti-Cullin 4B | Sigma-Aldrich | Cat# HPA011880; RRID:AB_1847340 | 1:200 |  |  |
| Mouse monoclonal anti-DDB1 (clone 8) | BD Biosciences | Cat# 612488; RRID:AB_399789 | 1:1000 |  |  |
| Rabbit anti-ERCC1 (clone FL-297) | Santa Cruz Biotechnology | Cat# sc-10785; RRID:AB_2278022 | 1:1000 | 1:500 |  |
| Rabbit monoclonal anti-FANCD2 (clone EPR2302) | Abcam | Cat# ab108928; RRID:AB_10862535 | 1:5000 | 1:500 |  |
| Mouse monoclonal anti-GAPDH | Abcam | Cat# ab9484; RRID:AB_307274 | 1:40'000 |  |  |
| Mouse monoclonal anti-Histone H2A.X phospho S139) (slone JBW301) | Millipore | Cat# 05-636; RRID:AB_309864 |  | 1:1000 |  |
| Mouse monoclonal anti-Histone H3 (phospho S10) (clone K.872.3) | Thermo Fisher Scientific | Cat# MA5-15220; RRID:AB_11008586 |  |  | 1:100 |
| Rat monoclonal anti-RPA2 (clone 4E4) | Cell Signaling Technology | Cat# 2208; RRID:AB_2238543 | 1:1000 | 1:200 |  |
| Rabbit polyclonal anti-RPA2 (phospho S33) | Novus | Cat# NB 100-544; RRID:AB_526631 | 1:5000 | 1:1000 |  |
| Rabbit polyclonal anti-RPA2 (phospho S4 + S8) | Abcam | Cat# ab87277; RRID:AB_1952482 | 1:1000 | 1:500 |  |
| Rabbit polyclonal anti-XPA (clone FL-273) | Santa Cruz Biotechnology | Cat# sc-853; RRID:AB_632612 | 1:100 |  |  |
|  |  |  |  |  |  |
| **WB**, Western blot; **IF**, Immunofluorescence; **FC**, Flow cytometry | | |  |  |  |
